# Does high biodiversity reduce the risk of Lyme disease invasion?

**DOI:** 10.1186/1756-3305-6-195

**Published:** 2013-07-01

**Authors:** Catherine Bouchard, Guy Beauchamp, Patrick A Leighton, Robbin Lindsay, Denise Bélanger, Nick H Ogden

**Affiliations:** 1 Groupe de recherche en épidémiologie des zoonoses et santé publique, Faculté de médecine vétérinaire, Université de Montréal, 3200 Sicotte, C.P. 5000, Saint-Hyacinthe, Québec, J2S 7C6, Canada; 2 Zoonotic Diseases and Special Pathogens, Public Health Agency of Canada, National Microbiology Laboratory, Winnipeg, MB, Canada; 3 Zoonoses Division, Centre for Food-borne, Environmental and Zoonotic Infectious Diseases, Public Health Agency of Canada, St-Hyacinthe, Québec, Canada

**Keywords:** *Ixodes scapularis*, *Borrelia burgdorferi*, Lyme, Host, Biodiversity, Invasion

## Abstract

**Background:**

It has been suggested that increasing biodiversity, specifically host diversity, reduces pathogen and parasite transmission amongst wildlife (causing a “dilution effect”), whereby transmission amongst efficient reservoir hosts, (e.g. *Peromyscus* spp. mice for the agent of Lyme disease *Borrelia burgdorferi*) is reduced by the presence of other less efficient host species. If so, then increasing biodiversity should inhibit pathogen and parasite invasion.

**Methods:**

We investigated this hypothesis by studying invasion of *B. burgdorferi* and its tick vector *Ixodes scapularis* in 71 field sites in southeastern Canada. Indices of trapped rodent host diversity, and of biodiversity of the wider community, were investigated as variables explaining the numbers of *I. scapularis* collected and *B. burgdorferi* infection in these ticks. A wide range of alternative environmental explanatory variables were also considered.

**Results:**

The observation of low *I. scapularis* abundance and low *B. burgdorferi* infection prevalence in sites where *I. scapularis* were detected was consistent with early-stage invasion of the vector. There were significant associations between the abundance of ticks and season, year of study and ambient temperature. Abundance of host-seeking larvae was significantly associated with deer density, and abundance of host-seeking larvae and nymphs were positively associated with litter layer depth. Larval host infestations were lower where the relative proportion of non-*Peromyscus* spp. was high. Infestations of hosts with nymphs were lower when host species richness was higher, but overall nymphal abundance increased with species richness because *Peromyscus* spp. mouse abundance and host species richness were positively correlated. Nymphal infestations of hosts were lower where tree species richness was higher. *B. burgdorferi* infection prevalence in ticks varied significantly with an index of rates of migratory bird-borne vector and pathogen invasion.

**Conclusions:**

*I. scapularis* abundance and *B. burgdorferi* prevalence varied with explanatory variables in patterns consistent with the known biology of these species in general, and in the study region in particular. The evidence for a negative effect of host biodiversity on *I. scapularis* invasion was mixed. However, some evidence suggests that community biodiversity beyond just host diversity may have direct or indirect inhibitory effects on parasite invasion that warrant further study.

## Background

The agent of Lyme disease in the Northeast and Midwestern North America, *Borrelia burgdorferi sensu stricto* (hereafter referred to as *B. burgdorferi*), is transmitted by the blacklegged tick, *Ixodes scapularis* (Acari: Ixodidae) [1-4]. The risk of Lyme disease is increasing in southern parts of eastern and central Canada due to *I. scapularis* ticks that are expanding their geographic range northwards through dispersion by songbirds during spring migration and facilitation of the establishment of tick populations by a warming climate [5-7]. These factors are likely common drivers of tick species range expansion in temperate zones, particularly when combined [8]. In the following we use the term “biodiversity” for the species diversity occurring in the whole biotic community of a site. When focussing on species diversity within particular groups we use the term “diversity”.

Invasion by non-native species, including pathogens, is frequently considered to have a direct or indirect negative effect on biodiversity in invaded ecosystems [9-12]. However, host species diversity has been hypothesised as a factor that reduces the transmission of micro- and macro-parasites, and this possible effect has been termed the ‘dilution effect’ [13-18]. The infectious disease transmission system for which the effects of biodiversity have been most examined is that of *B. burgdorferi*[19]. However, convincing field evidence of host diversity diminishing Lyme disease transmission cycle occurrence is, in the eyes of some scientists, limited [19-21]. Here we hypothesise that if biodiversity is a factor that reduces transmission of *B. burgdorferi* and other pathogens and parasites, it should also be a factor that inhibits (i.e. slows or prevents) invasion of pathogens and parasites into a new location. *Ixodes scapularis* ticks, followed by *B. burgdorferi*, are currently invading southeastern and south central Canada offering us a unique opportunity to investigate whether or not biodiversity has an effect on the invasion of these species.

Previously, the effect of host diversity has been considered as a factor affecting parasite and pathogen transmission cycles. *B. burgdorferi* circulates in an enzootic cycle between *I. scapularis* ticks and mammalian and avian tick and/or reservoir hosts [22]. *Peromyscus leucopus*, the white-footed mouse, has frequently been considered as a key reservoir host for *B. burgdorferi* in North America and this seems to be true for sites in southern Quebec, Canada [23]. Due to the host generalist behaviour of *I. scapularis*, increasing diversity of animal host species increases the possibility that ticks are diverted away from *P. leucopus* onto animals that are less efficient reservoirs of *B. burgdorferi*, which could result in a dilution effect [24]. However, the increased abundance of hosts associated with increasing biodiversity would likely boost abundance of ticks (which are obligate parasites at all feeding life stages), which calls into question the existence of a consistent inverse relationship between host biodiversity and tick and tick-borne pathogen abundance [25,26].

If increasing biodiversity does inhibit invasion by reducing pathogen and parasite transmission amongst hosts, then in the zone of *B. burgdorferi* and *I*. *scapularis* invasion in southern Quebec, tick abundance and *B*. *burgdorferi* infection prevalence in ticks should be lower in locations where biodiversity is higher. We investigated possible effects of host diversity, but we also considered the more holistic view that biodiversity may impact on tick survival and pathogen transmission cycles by mechanisms involving competitors, predators and pathogens/parasites (for example) that are independent of host species diversity. In particular we explored direct measures or indices of vertebrate host diversity, but also indices such as tree species richness that may act as proxy measures of biodiversity of the wider community, as factors associated with tick and *B*. *burgdorferi* occurrence.

At the same time, a range of other environmental factors that are known to influence tick-borne disease transmission and invasion of ticks and tick-borne pathogens needed to be taken into consideration. These included i) inter-site variations in abiotic environmental factors such as climate, elevation, aspect, drainage and slope that affect tick survival [5,27-29]; and ii) inter-site variations in the rate of import of ticks and bacterium on/in migratory birds [6] or terrestrial hosts from neighbouring or more distant source locations where the invading ticks and tick-borne pathogens are already established [30]. In addition, our findings need to be interpreted in light of the likely unequilibrial population dynamics of ticks and *B. burgdorferi* as these organisms become established [31].

## Methods

### Study sites

**Figure 1:**
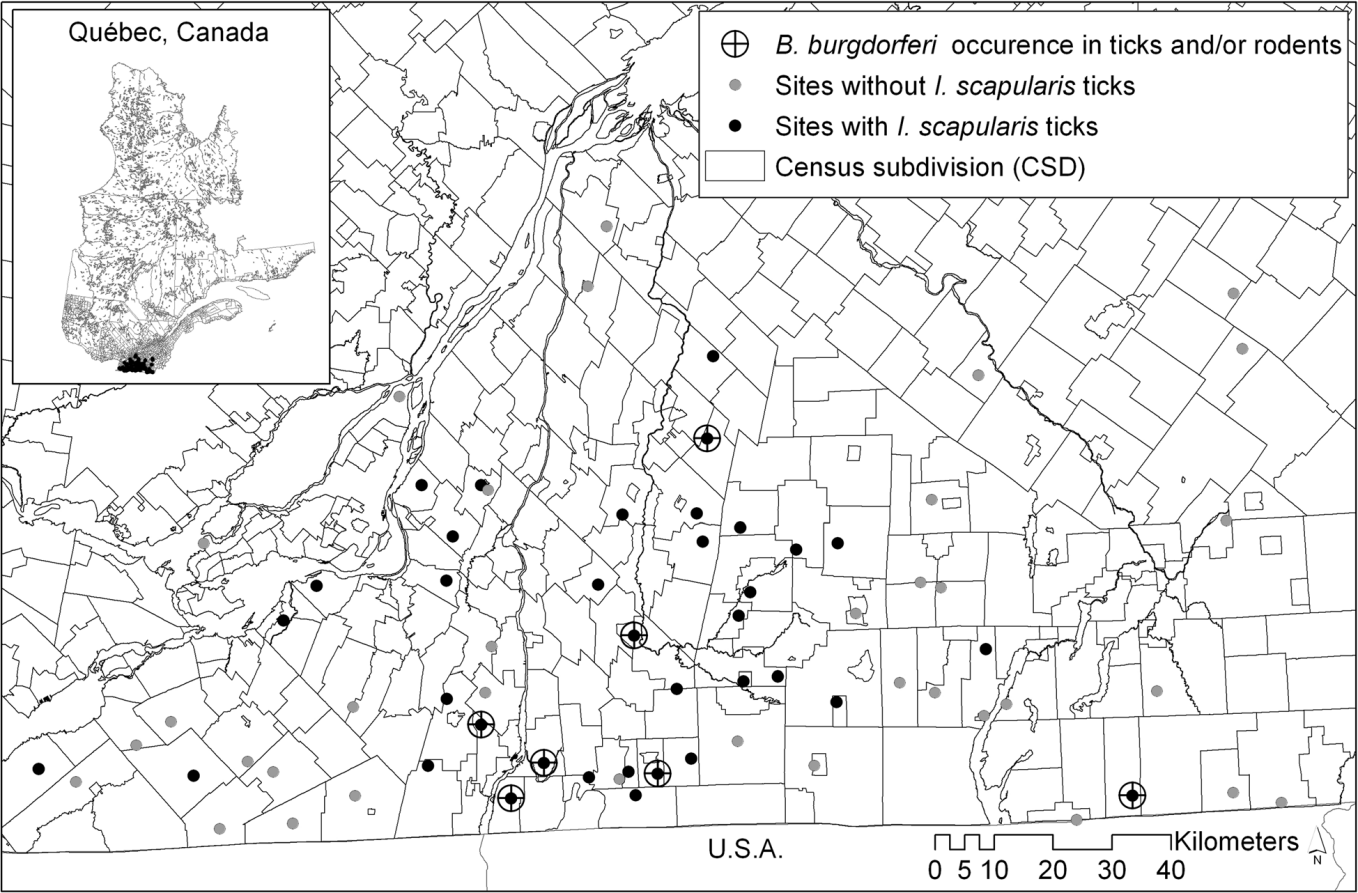
***Ixodes scapularis *and *B. burgdorferi *occurrence at the 71 sites in southwestern Quebec, 2007–2008.**

The study was carried out in southwestern Quebec at 71 sites: 46 sites were visited in 2007 and an additional 25 sites were visited in 2008. The 71 sites were located in three different regions of Quebec (Montérégie, Estrie and Montréal) covering 5,325 km^2^ (Figure 1). Some sites were visited once only either in 2007 and 2008 (one single visit per site from June to October in 2007 or from May to October in 2008), but some sites visited in 2007 were revisited in 2008 (see [23] for details).

Sites were selected on the basis of comprising deciduous (maple or mixed deciduous) woodland [32] of minimal dimensions 500 m by 150 m, with ease of access [7,23]. Sampling was conducted within a 75,000 m^2^ (150 m × 500 m) trapping grid.

### Collection and sampling of ticks and captured mammals

Host-seeking ticks were collected by a standard effort of 3 person-hours of dragging a 1 m^2^ white cotton flannel sheet using a standard pattern (drags occurring parallel to, and either side of, each trap transect) within the trapping grid at each site visit. Drag sampling did not occur during periods of heavy rainfall.

At each site, 150 Sherman™ live traps were placed in three parallel transects of 50 traps each for one or two consecutive days and nights. Traps were placed for two nights if fewer than 15 *Peromyscus* spp. mice (white-footed mice and deer mice) were captured on the first night. This sample size was required to give adequate statistical power for the study objectives of determining the geographic scope of establishment of *B. burgdorferi* and *I. scapularis* ticks in southwestern Quebec (for details see [23]). Feeding ticks were collected from trapped rodents according to a previously described protocol [23].

Following capture, animals were lightly anaesthetised as previously described [23] and then thoroughly examined for ticks. Any ticks found were collected into tubes containing 75% ethanol. Blood was collected from each *P. leucopus* mouse with a 23 gauge needle and syringe directly from the heart, or via the infra orbital sinus using 150 μl Natelson™ blood collecting tubes, and placed in sample tubes containing EDTA. In 2008, eastern chipmunks and red squirrels were also bled for serological analyses since more than 10% of *I. scapularis* were found on these species in 2007. All procedures were undertaken with appropriate ethical approval by the Ministère des Ressources naturelles et de la Faune of the Province of Quebec (MRNF) and the Université de Montréal (see [23] for further details).

### Testing of tick and rodent samples for *B. burgdorferi* infection

Ticks were identified using standard keys [33,34] and DNA was extracted from *I. scapularis* ticks and tested for the presence of *B. burgdorferi* by polymerase chain reaction (PCR)*.* DNA was obtained using Qiagen® DNeasy® 96 Tissue kits (QIAGEN Inc., Mississauga, ON, Canada) optimized for recovery of low-copy number DNA from ticks, and extraction efficiency was assessed using primers specific for the tick 5.8S rRNA - 28S rRNA intergenic spacer (IGS). DNA was screened for evidence of *B. burgdorferi* infection using a multiplex real-time PCR targeting the 23S rRNA of *B. burgdorferi* as previously described [35]. *B. burgdorferi* infection was then confirmed in positive samples using primers targeting the *ospA* gene [5].

Plasma was separated from the blood samples by centrifugation at 2,000 g for 6 minutes and kept frozen (−20°C) until shipment on dry ice to the National Microbiology Laboratory (NML) for testing. All plasma samples were tested for IgG antibodies to *B. burgdorferi* using an in-house immunofluorescent assay, followed by a Western blot on reactive samples in 2007, and a two-tier ELISA and Western blot assays, adapted for testing rodent sera, in 2008, as previously described [36].

#### Collection and development of explanatory variables

##### Small mammal hosts

For each location and visit, the number of captured *Peromyscus* mice (*P. leucopus* and *P. maniculatus* combined), total numbers of rodent captures, rodent species richness, Shannon diversity index [37] and the relative proportion of captured rodents that were *Peromyscus* species were used as covariates.

##### White-tailed deer hosts

Since deer are essential hosts for adult *I. scapularis* and also host immature *I. scapularis*, their density would be expected to influence *I. scapularis* density [38]. The density of white-tailed deer was estimated from harvested male deer distributions obtained from the MRNF database for 2007 and 2008 (http://ftp://ftp.mrnf.gouv.qc.ca/Public/Defh/Publications/Archives/Daigle%202007_Rapport-syst-suivi.pdf) (http://www.mddefp.gouv.qc.ca/faune/publications/chasse/plan-gestion-cerf-2010-17.pdf). We extracted a deer density estimate at each different site location based on these data. Deer abundance estimates from aerial censuses are positively correlated with hunted male deer estimates used in this study area (R^2^ = 0.84) [39].

##### Abiotic environmental factors

Climatic conditions, particularly ambient temperature, have been recognised as a consistently important determinant of where *I. scapularis* can become established in Canada, during modelling studies, field validation of model outcomes, and analysis of surveillance data [7,40-42]. Therefore, climate is a factor that needs to be accounted for in any assessment of the impact of biodiversity on invasion by *I. scapularis*.

To do this, temperature and precipitation data were obtained from Environment Canada for 32 southwestern Quebec meteorological stations within 50 km^2^ of the centre of the study region that reported data during 2003–2008 (http://climate.weatheroffice.gc.ca/prods_servs/index_e.html). Temperatures above 0°C were summed for each day of the year to obtain annual accumulated degree days > 0°C (DD > 0°C which is a useful index of temperature suitability for *I. scapularis*:) at each climate station in southern Quebec and an interpolated surface of DD > 0°C was obtained from these data using inverse distance weighting (IDW) as previously described [7,40]. From the interpolated surfaces, we extracted a value of mean annual DD > 0°C for each site. Total annual rainfall (mm) for each site was obtained using the same interpolation method.

Soil type (based on percentage of sand, clay and organic matter: [43]), and site aspect (categorized as 1: hill crest, 2: upper slope, 3: middle slope, 4: lower slope, 5: toe [land immediately beyond the foot of a slope], 6: depression, 7: level ground, and 8: complex [a mix of the previous]) were assessed and recorded on the first site visit. Site slope gradient (gradient based on slope %) and index of soil drainage at each site (nil to very good drainage) were obtained from 3rd or 4th inventory of Système d’information écoforestière (SIEF) of MRNF at a scale of 1:20,000 (http://www.mrnf.gouv.qc.ca/forets/connaissances/connaissances-inventaire-cartes-sief.jsp). These variables would be expected to most strongly affect the environment of off-host ticks undergoing development in the litter layer, affecting mortality of these ticks as a consequence of dehydration in very dry habitats or drowning in very wet habitats [41,44].

##### Biotic environmental factors

Species richness of mature trees and the understory herbs and shrubs were assessed on the first site visit: the species of all trees and shrubs within, or overhanging, the trapping grid were identified, and as many herbs as possible were identified within a 2 hour period. In addition values for litter depth (in cm), tree population age, tree height (in m) and density, and the patch size (in km^2^) of the forest within which the site occurred were obtained from the SIEF inventories (http://www.mrnf.gouv.qc.ca/forets/connaissances/connaissances-inventaire-cartes-sief.jsp). The patch size might be an indirect measure of rodent host density and host diversity [45,46], while forest inventory metrics of tree age, height and density may acts as proxy indices of woodland maturity, degree of disturbance, biodiversity [47,48] and more general habitat classifications act as indices of the suitability of habitat for off-host tick survival [41]. Values and ranges for all biotic and abiotic variables are presented in Additional file 1: Table S1 in supplementary on-line information.

#### *Accounting for rates of* I. scapularis *and* B*.* burgdorferi *immigration*

##### Introduction via migratory birds

Northward migrating passerines in spring are thought to be a significant route for introduction of *I. scapularis* and *B. burgdorferi* into southern Canada. The timing and routes of migration mean that these birds acquire questing ticks (particularly nymphs, which may be infected with *B. burgdorferi*) in locations in the northeast and mid-west of the USA where *I. scapularis* and *B. burgdorferi* are established. Attached nymphal ticks can then be dispersed north into and across Canada for distances of 450 km or more [6,7]. With knowledge of the main geographic locations in the USA and Canada where *I. scapularis* and *B. burgdorferi* are established we have developed an index of the numbers of immigrating bird-borne ticks (the ‘adventitious tick index’) for southern Canada in general [6] and for each of the 71 study sites in particular [42,49]. This index is effectively also an index of rates of introduction of *B. burgdorferi* by infected migratory birds or the ticks they carry.

##### Introduction by terrestrial hosts

We do not have any indices of rates of movement of *I. scapularis* or *B. burgdorferi* by terrestrial hosts. However, we assumed that if rates of immigration of ticks or bacterium in/on terrestrial/resident hosts were having a significant impact on the observed pattern of *I. scapularis* or *B. burgdorferi* occurrence amongst the study sites, the spatial pattern of establishment of *I. scapularis* or *B. burgdorferi*[49] would be reflected in significant spatial autocorrelation [50]. The possible presence of spatial autocorrelation (which would in any case violate the assumption of independence of observations for all analyses), was explored in post-hoc analyses of residuals using semi-variograms plotted as a function of geographic distance [51].

### Statistical analyses

We used R software v.2.13.2 (R development Core Team, 2008) for all statistical models, which accounted in all cases for year of study (2007 versus 2008) and season (‘spring’ being April to June and ‘summer’ being July to October), amongst which tick and mammal abundance vary.

The following models were developed:

Models 1–4. Models investigating factors affecting *I. scapularis* abundance: Given a high level of overdispersion of the questing and feeding tick counts, generalized linear models (GLM) with a negative binomial distribution were used to model tick abundance following elimination of zero-inflated Poisson and zero-inflated negative binomial models as alternatives on the basis of best fit (determined on the basis of the lowest AICc). Four outcomes were explored: i) abundance of questing larvae, ii) abundance of questing nymphs, iii) abundance of feeding larvae, and iv) abundance of feeding nymphs. All explanatory variables were used in all models, however, the number of captured mammals was also included as an offset in the latter two.

Model 5. A model investigating factors affecting the abundance of *Peromyscus* mice: A linear regression model was constructed to investigate factors explaining the numbers of *Peromyscus* mice captured. All environmental and host explanatory variables were used in the model, with the exception of the number of *Peromyscus* mice captured.

Model 6. A model investigating factors associated with *B. burgdorferi* infection in feeding ticks. A logistic regression model was developed with the *B. burgdorferi* PCR result of feeding ticks as the outcome. Infection in questing ticks was not investigated as few of these were positive at the time of sampling. All environmental and host variables were explored.

In all models site ID was treated as a random effect. In each model, explanatory variables were tested individually with a liberal cut-off of P < 0.2 in a multivariable model and then we selected the most parsimonious multivariable model through a process of forward and backward substitution and elimination. The cut-off for keeping a variable in the final model was P < 0.05. To account for possible multicollinearity between covariates, we set a threshold of 3 for variance inflation factors [52].

## Results and discussion

### Tick abundance and *B. burgdorferi* infection

Details of the numbers of ticks and rodents collected are presented in previous work [31] and tick data are summarised in Table 1. *Ixodes scapularis* ticks were found on 37 different sites by flagging and/or examination of trapped mammals. Of 1278 *I. scapularis* ticks collected, 932 were larvae, 309 were nymphs and 37 were adults. Almost all *I. scapularis* ticks were found in Montérégie region. Nymphal *I. scapularis* were found at 28 of the 71 sites. At all but one of the sites where *I. scapularis* were found in 2007, at least one tick was found when we revisited the sites in 2008. Overall, we found immature *I. scapularis* ticks at 34 sites (2 instars at 13 sites and 3 instars at 8 sites) and adult ticks only at 11 sites [40]. Evidence of transmission of *B. burgdorferi* (PCR-positive ticks or seropositive rodents) was found at 7 sites (Figure 1). The prevalence of infection in ticks was 1.8–3.3% for feeding larvae (12–22 of 675 tested; 11 larval ticks from one rodent were pooled and the pool tested positive), 0.7% (1 of 135 tested) for questing nymphs, 9.9% (17 of 172 tested) for feeding nymphs, and 5.6% (2 of 36) for questing adults (Table 1). Questing larvae were not tested because *B. burgdorferi* is not transmitted from female *I. scapularis* to their progeny [53].

**Table 1:** **Number and *B. burgdorferi *infection status (Bb+) of questing and feeding ticks in three regions of southwestern Quebec, 2007-2008**

**Region**	**No. of sites**	**No. of visits**	**Larvae**		**Nymphs**		**Adults**		**Total (Bb+)**
			**QL**^**a**^	**FL (Bb+)**	**QN (Bb+)**	**FN (Bb+)**	**QA (Bb+)**	**FA (Bb+)**	
Estrie	16	17	0	0 (0)	0 (0)	2 (0)	0 (0)	0 (0)	2 (0)
Montérégie	53	83	251	681 (22)	136 (1)	171 (17)	36 (2)	1 (0)	1276 (42)
Montréal	2	2	0	0 (0)	0 (0)	0 (0)	0 (0)	0 (0)	0 (0)
Total	71	102	251	681 (22)	136 (1)	173 (17)	36 (2)	1 (0)	1278 (42)

^a^ Questing larvae were not tested for *B. burgdorferi* infection.

Together these findings indicated that *I. scapularis* was establishing in sites in southern Quebec, but that the tick and bacterium may be at a relatively early stage of establishment in terms of the abundance of ticks and the prevalence of *B. burgdorferi* infection. Modelling studies suggest that while maximum tick population densities at any particular location may be limited or regulated by density-dependent acquired host resistance, it will take a number of population cycles for tick abundance to approach the potential maximum for that location and for density-dependent mechanisms to operate. Therefore, any observed effect of host diversity on *I. scapularis* occurrence in our study would likely operate more through innate factors (e.g. innate host resistance and grooming) than through acquired resistance to ticks [54]. Modelling studies also identify low tick abundance as a limiting factor on invasion of *B. burgdorferi* and the prevalence of infection in ticks and hosts [54,55].

### Associations of explanatory variables with the abundance of *I. scapularis* and *B. burgdorferi* occurence

Details of statistical model results are presented in Tables 2, 3, and 4.

**Table 2:** The variables, parameter estimates (β), standard errors (SE), z-values and p-values for two negative binomial generalized linear models of the count of questing and feeding ticks collected in southwestern Quebec, 2007-2008

**Model**	**Outcome**	**Explanatory variables**		**β**	**SE**	**z-value**	**p-value**
Model 1	Count of questing larvae	*Year*	(2008 vs 2007)	2.18	0.97	2.25	0.02
		*Litter depth*		0.39	0.13	3.01	< 0.01
		*Temperature*		0.01	0.01	2.65	< 0.01
		*Deer density*		0.92	0.16	5.83	< 0.01
Model 2	Count of questing nymphs	*Year*	(2008 vs 2007)	1.72	0.55	3.15	< 0.01
		*Litter depth*		0.22	0.07	3.04	< 0.01
		*Temperature*		0.01	0.00	2.41	0.02
Model 3	Count of feeding larvae	*Proportion of trapped mammals that were Peromyscus* spp.		1.98	0.99	1.99	0.05
		*Site gradient*		−1.68	0.55	−3.05	< 0.01
		*Drainage*		0.11	0.04	2.68	< 0.01
		*Height of mature trees*		0.42	0.21	2.06	< 0.01
		*Temperature*		1.46	0.43	3.42	< 0.01
		*Deer density*		1.10	0.23	4.80	< 0.01
Model 4	Count of feeding nymphs	*Year*	(2008 vs 2007)	1.05	0.01	10.75	< 0.01
		*Season*	(Summer vs spring)	−0.50	0.12	4.31	< 0.01
		*Species richness of rodents*		−0.18	0.05	−3.49	< 0.01
		*Site gradient*		−0.69	0.09	−8.19	< 0.01
		*Species richness of mature trees*		−0.04	0.02	−2.49	0.01
		*Density of mature trees*		−0.33	0.08	−4.34	< 0.01
		*Temperature*		0.33	0.10	3.25	< 0.01

**Table 3:** **The variables, parameter estimates (β), standard errors (SE), t-values and p-values for a linear regression model of the count of *Peromyscus *species captured in southwestern Quebec, 2007-2008**

**Model**	**Outcome**	**Explanatory variables**		**β**	**SE**	**t-value**	**p-value**
Model 5	Numbers of *Peromyscus* mice captured	*Season*	(Summer vs spring)^a^	7.34	1.20	6.10	< 0.01
		*Species richness of rodents*		1.38	0.60	2.28	0.02

^a^ Summer months: August, September, October vs Spring months: May, June, July.

**Table 4:** **The variables, parameter estimates (β), standard errors (SE), t-values and p-values for a logistic regression model of the occurrence of *B. burgdorferi *infection in feeding ticks collected in southwestern Quebec, 2007–2008**

**Model**	**Outcome**	**Explanatory variables**	**β**	**SE**	**t-value**	**p-value**
Model 6a (feeding larvae)	*B. burgdorferi* infection status	*Adventitious tick index*	1.21	0.39	3.12	< 0.01
Model 6b (feeding nymphs)	*B. burgdorferi* infection status	*Adventitious tick index*	2.03	0.93	2.17	0.03

### Abiotic environment factors

As anticipated, temperature conditions were significantly associated with the abundance of ticks in each of models 1–4, which is consistent with all recent studies on *I. scapularis* in Canada [7,40,49]. *I. scapularis* were more abundant in 2008 than 2007 while accounting for site in the models (most sites with *I. scapularis* in 2007 were re-visited in 2008), consistent with increasing abundance of ticks as their populations become more firmly established. Season was a significant factor for infestations of hosts with nymphs (which were greater in spring: Table 2) and numbers of captured *Peromyscus* spp. mice (which were greater in summer: Table 3). Both of these findings are consistent with the known seasonal activity pattern of *I. scapularis* and seasonal variations in *Peromyscus* spp. densities in the region [31]. In northeastern North America, nymphal *I. scapularis* are most abundant in spring (due to their lifecycle and the suitability of spring weather for tick activity), while *P. leucopus* abundance is lowest in spring, and then increases in late summer/autumn due to recruitment of young mice [55].

Steeper site gradients were negatively associated with infestations of rodents with feeding larvae and nymphs, suggesting that steeper gradients provide less suitable habitats for ticks [56]. Increased drainage was associated with increased infestation levels, suggesting greater survival in the sites with greater drainage consistent with studies in the USA [57], but particularly relevant in our study area where many woodlands regularly flood in the spring [44] (http://foliogis.ducks.ca/qc/fr/monteregie/reg16_rapport_avril08.pdf).

### Biotic environment and biodiversity-associated factors

Litter depth was positively associated with numbers of larvae and nymphs collected by dragging. This is consistent with increased leaf litter providing a more ‘insulated’ refuge that better protects ticks from extremes of temperature and desiccation thus promoting their survival (e.g. [58]). However, litter depth was not associated with abundance of feeding larvae and nymphs so the possibility that litter depth simply altered the ability of dragging to collect ticks (perhaps by affecting how high ticks quest from the woodland floor) cannot be ruled out.

Habitats with higher densities of white-tailed deer had more host-seeking larvae and more larva per host rodent but interestingly the latter was not true for nymphs, when deer are hosts for both larvae and nymphs in proportions similar to rodents [59]. This observation is consistent with deer being the principal hosts for adult female ticks, which after feeding would drop from these hosts and produce eggs that give rise to the subsequent cohort of larvae. Clearly nymphal abundance depends on larval abundance and thus is indirectly dependent on deer abundance, but it is not surprising that deer density may have a less detectable association with nymphal abundance as there is considerable mortality between larval and nymphal instars which results from a wide range of deer-independent factors. Indeed, the ratio of feeding larvae to feeding nymphs on the rodents was approximately 8 to 1 after accounting for the approximately two-fold difference in duration of feeding of larvae and nymphs. Higher abundance of larvae than nymphs, and the dependence of larval numbers on deer abundance alone rather than on the range of hosts on which nymphal numbers depend, may explain why indices of host diversity were associated with feeding nymphal tick numbers, but not feeding larval tick numbers in the following.

Infestations of rodents with feeding larvae were lower on sites where the relative proportion of *Peromyscus* spp. mice versus other rodents was lower, consistent with Bouchard *et al*. (2011) where we identified that *Peromyscus* spp. mice carried the majority of larvae on our study sites. This could be due to greater contact rates with questing larvae by virtue of mouse behaviour and habitat use, ineffective innate or acquired immune responses to ticks, and/or less effective grooming compared to other species [31]. In addition, infestation of rodents with nymphal *I. scapularis* was lower on sites with higher species richness. These observations suggest that higher relative abundance of *Peromyscus* spp. favours *I. scapularis* invasion, but it does not directly support a hypothesis of host diversity reducing risk of tick invasion because the density of *Peromyscus* spp. on the sites (assuming numbers captured equate with density) increased with species richness (Table 3). This latter observation is somewhat counterintuitive considering the general perception that *P. leucopus* mouse abundance (which were the most abundant *Peromyscus* species on our sites) is higher in disturbed and fragmented habitats that are considered to favour these generalist species and to be associated with lower host diversity [2,60]. So, where species richness is greater, nymphal infestations were lower, but *Peromyscus* mouse abundance was higher, which raises the question, what is the net effect of increasing rodent species richness (to the maximum 10 species in the study [23]) on feeding nymph densities on the sites? We estimate that the effect on feeding nymph abundance, of increasing species richness by one species, would be a reduction in 1.76 feeding nymphs per site using the following simple equation:

$$\mathrm{dN}=e^{\mathrm{cnb}}*\mathrm{Nr}*R$$

where *dN* is the decrease in the number of feeding nymphs per site, *cnb* is the coefficient (−0.18) for the effect of change in rodent richness in the negative binomial regression model for feeding nymphs (Model 4 in Table 2), *Nr* is the mean number of nymphs per rodent (173/1 295 = 0.133), and *R* is the median number of rodents per observation (18).

In contrast, the effect of increasing species richness by one species on increasing nymph abundance, via effects of species richness on *Peromyscus* spp. abundance, was estimated as 1.85 using the following equation:

$$\Delta N=N_{p}*\mathrm{cr}*P$$

Where *ΔN* is the increase in the number of feeding nymphs per site, *Np* is the mean number of nymphs per *Peromyscus* mouse (98/810 = 0.121), *cr* is the coefficient for the effect of change in rodent richness in the regression model for *Peromyscus* capture numbers (Model 5, Table 3), and *P* is the median number of *Peromyscus* spp. mice per observation (11).

Combining these two opposite effects, increased nymphal abundance due to increasing *Peromyscus* spp. mouse density with greater species richness would more than compensate for the concomittant decreased host infestation levels and result in an overall (albeit small) increase in abundance of feeding nymphs on the sites with higher host diversity. A caveat to this analysis is that the full host community that includes birds and medium sized animals was not assessed (for operational necessity) and included.

There was evidence that increases in overall community biodiversity, beyond just rodent host diversity, may inhibit tick survival and invasion: richness of mature tree species was significantly and negatively associated with nymphal infestation levels (Table 2). Other significant environmental variables include mature tree height (positively associated with larval tick infestations) and mature tree density (negatively associated with nymphal tick infestations) (Table 2). Both of the latter variables could be proxies for particular types of habitat and be indirect measures of host abundance or off-host tick survival. Tree density has been identified as being correlated with biodiversity in one study (Paquette & Messier 2011). Therefore, further exploration of the associations between tree species richness, tree height, tree density, community biodiversity and tick occurrence and survival are needed.

### Variables associated with *I. scapularis* and *B. burgdorferi* immigration

Abundance of *I. scapularis* of any stage was not detectably associated with our proxies for rates of immigration. This finding is consistent with the abundance of ticks on *I. scapularis*-positive sites being due to intrinsic factors affecting tick reproduction and survival in newly-established tick populations rather than relatively low numbers of immigrating ticks, such as migratory bird-borne ticks [61]. However, the ‘adventitious tick index’, as a proxy of variation in rates of immigration of *B. burgdorferi* amongst sites, was the only factor significantly associated with infection in engorged larvae and nymphs, suggesting that rates of immigration of *B. burgdorferi* rather than factors associated with site-specific transmission were the most important determinants of infection levels at the time the study took place. This is consistent with our knowledge of the current status of ticks and *B. burgdorferi* invasion in the region and underlines the dynamic non-equilibrial status of tick and *B. burgdorferi* populations in invasion zones [31]. In turn this highlights the need for more longitudinal studies in sentinel sites to understand the temporal dynamics of invasion of tick-borne pathogens.

## Conclusions

There was remarkable consistency of model outcomes with the known biology of *I. scapularis* and *B. burgdorferi* in general, and particularly in the region where these species are invading in southern Canada. This provides confidence in the methods and results, and of our capacity to correctly interpret the latter.

Increasing host diversity was associated with reduced nymphal tick infestations of rodent hosts, which at first sight could be interpreted as evidence of a dilution effect inhibiting invasion of *I. scapularis*. However, increased host diversity was associated with higher *Peromyscus* spp. mouse abundance, and the increase in nymphal abundance associated with increasing mouse abundance was estimated to more than account for reduced individual host nymphal tick infestations. Therefore, overall, feeding nymphal tick abundance was positively correlated with host species richness, providing little support for a hypothesis that increasing host diversity inhibits *I. scapularis* invasion through the mechanism of the dilution effect.

In contrast, there was an association between tree species diversity and reduced host nymphal tick infestations, which did not affect *Peromyscus* spp. mouse abundance. This suggested a possible inhibitory effect of a more diverse biotic community (beyond simply the vertebrate host community) on *I. scapularis* invasion. Further study is needed to confirm if this association is a directly mechanistic effect of biodiversity or a proxy for other environmental effects on tick survival. Variations in *B. burgdorferi* infection prevalence in ticks was associated with an index for rates of invasion rather than local site characteristics, supporting the assumption that *B. burgdorferi* invasion in the study region was at an early stage at the time of sampling. This reinforces the need for longitudinal studies at sentinel sites to better understand pathogen and parasite invasion processes.

## Competing interests

The authors have no competing interests.

## Authors’ contributions

CB performed field study and data analysis in collaboration with GB, NHO, PAL and DB. NHO designed the study with LRL. LRL performed laboratory analyses. CB and NHO led manuscript preparation to which all authors subsequently contributed and approved.

## Supplementary Material

Additional file 1: Table S1 Description of environmental variables that were used in the statistical models. EC = Environment Canada. Click here for file
