# Supplementary material for: Does high biodiversity reduce the risk of Lyme disease invasion?
Source: Parasit Vectors. 2013 Jul 1;6:195. doi: 10.1186/1756-3305-6-195 (PMC3728044; doi:10.1186/1756-3305-6-195)
Supplement: Additional file 1: Table S1 — Description of environmental variables that were used in the statistical models. EC = Environment Canada. [file 1756-3305-6-195-S1.docx]

Table S1. Description of environmental variables that were used in the statistical models. EC = Environment Canada.

| **Environmental variables** | **Units of measure** | **Mean** | **Median** | **SE** | **Range** | **Source** |
| --- | --- | --- | --- | --- | --- | --- |
| *Small mammals* | | | | | | |
| Numbers of *Peromyscus* mice | count | 12.74 | 13 | 7.24 | 0-28 | Site |
| Proportion of *Peromyscus* mice | % | 59.00 | 62.00 | 23.60 | 0-100 | Site |
| Total numbers of rodents | count | 21.28 | 19 | 10.90 | 0-48 | Site |
| Species richness of rodents | count | 4.16 | 4.00 | 1.85 | 0-10 | Site |
| Shannon diversity index | low to high | 1.02 | 1.14 | 0.49 | 0-2.01 | Site |
| *White-tailed deer* | | | | | | |
| Deer density estimate | /km^2^ | 3.78 | 3.36 | 3.07 | 0.19-20.44 | [1] |
| *Migratory birds* | | | | | | |
| Adventitious tick index | low to high | 59.79 | 62.00 | 6.10 | 44-68 | [2] |
| *Habitat* | | | | | | |
| Forest patch size | km^2^ | 314.06 | 14.16 | 1357.73 | 0-6835.30 | [3] |
| Species richness of mature trees | count | 7.59 | 8 | 2.89 | 0-15 | [3] |
| Species richness of understory | count | 17.35 | 16 | 9.22 | 5-46 | [3] |
| Tree population age | year (categorized) |  | 30 |  | 10->100 at 10 year intervals | [3] |
| Tree height (dominant and codominant trees) | Category (m) |  | 2 (17-22m) |  | 1 (0m) - 7 (>22m) | [3] |
| Tree density | % covered (categorized) |  | 2 (61-81%) |  | 25% - >80% | [3] |
| *Climate* | | | | | | |
| Temperature (mean annual) | DD > 0°C | 3177.85 | 3215.95 | 152.83 | 2612.82-3341.25 | [4] |
| Precipitation (total rainfall) | mm | 1121.57 | 1120.58 | 86.83 | 981.09-1326.00 | [4] |
| *Soil/slope* | | | | | | |
| Proportion of sand | % | 14.55 | 8.00 | 17.00 | 0-85.00 | Site |
| Proportion of leaf mold | % | 53.75 | 55.00 | 26.07 | 0-100.00 | Site |
| Proportion of clay | % | 31.7 | 28.00 | 27.83 | 0-95.00 | Site |
| Litter depth | cm | 3.69 | 3.50 | 1.97 | 0.5-8.5 | Site |
| Site aspect | Category |  | 7 |  | 1-8* | Site |
| Slope gradient | Category |  | 1 (0-3%) |  | 1(0%)-6(>41%) | [3] |
| Index of soil drainage | Category |  | Moderate |  | Very poor, poor, moderate, good | [3] |

* Refer to methods for full description of these categories

**Data sources:**

1. <http://www.mrnf.gouv.qc.ca/cerfs>

2. Leighton PA, Koffi JK, Pelcat Y, Lindsay LR, Ogden NH: **Predicting the speed of tick invasion: an empirical model of range expansion for the Lyme disease vector *Ixodes scapularis in Canada***. *Journal of Applied Ecology* 2012, **49**(2):457-464.

3. <http://www.mrnf.gouv.qc.ca/forets/connaissances/connaissances-inventaire-cartes-sief.jsp>.

4. http://climate.weatheroffice.gc.ca/prods_servs/index_e.html
